# Decisions at the end of life: have we come of age?

**DOI:** 10.1186/1741-7015-8-57

**Published:** 2010-10-08

**Authors:** Linda Emanuel, Karen Glasser Scandrett

**Affiliations:** 1 Buehler Center on Aging, Health and Society, Northwestern University Feinberg School of Medicine, 750 N. Lake Shore Drive, Chicago, IL 60611, USA

## Abstract

Decision making is a complex process and it is particularly challenging to make decisions with, or for, patients who are near the end of their life. Some of those challenges will not be resolved - due to our human inability to foresee the future precisely and the human proclivity to change stated preferences when faced with reality. Other challenges of the decision-making process are manageable. This commentary offers a set of approaches which may lead to progress in this field.

One clearly desirable approach can and should be used more often than it is: the routine inclusion of discussions about the goals of care and documentation with all patients who have a poor prognosis. The match between a patient's goals and the care received should be the gold standard for quality palliative care.

Planning for future situations is necessary but hard. In order to achieve efficient elicitation and documentation of advance care planning, research is needed on each individual's thresholds for transitioning from curative to palliative intent and on the trajectory of changed preferences when illness occurs. Another clearly desirable approach is the documentation and use of community preferences, so that proxies making decisions without guidance from the patient can at least know what the majority of people considering similar situations chose to do.

Part of the challenge of achieving 'quality dying' may have to do with the still current (mainly Western) tendency to a death-denying culture and the inability of dying people to enter into the dying role. Awareness of the tasks of the dying role and the provision of time and space for those tasks during the delivery of medical care is essential. Medicine needs to continue to enhance the existential maturity of our profession, our patients and the cultures in which we practice. This state of mind should provide for decisions made with a more settled acceptance of mortality and with more awareness of the necessary connection to our survivors and next generation that mortality creates. Specific interventions, such as Dignity Therapy and advance care planning, may aid this state of mind.

## Introduction

Decision theory has become a specialized topic of interest and study within several disciplines: in medicine, decision theory draws on economics (especially game theory), moral philosophy, psychology, sociology and the law. The roots of decision-making are eternal and so critical that Greek mythology used it as a key theme. For instance, planning in advance to manage his own anticipated feelings Odysseus had himself tied to the mast to help him resist the Sirens [1]. Indeed, even further back in our evolutionary history, the effective execution of decision-making by individuals must have been quite refined, as many decisions directly determine immediate and long-term survival. Theorists and researchers who model and measure decision-making should not expect this to be an easy task. Human equipment for decision-making stems from multiple levels of consciousness: intuitive, driven by subconscious processes; gut level, driven by adrenal hormones; cognitive and uniquely rational; and cognitive but socially influenced in complex ways. Further, decisions are made and reviewed prospectively, in real time and in retrospect. Each of these features is not only complex but somewhat plastic and humanly fallible. Not only does decision-making have ancient roots, it also seems to represent something critical about human nature and human dignity: it is the central manifestation of autonomy and respect for a person's decision is often understood to be the equivalent of respect for that person as a whole.

Decision-making sciences have been troubled by several features. First, decisions are changeable. One striking example is that of women who decline pain management before giving birth but beg for it during labour underscores the reality [2]. Second, decisions are malleable, being influenced by others. Indeed the influence of the clinician on decisions is so critical that it defines the type of relationship models that exist in medicine [3]. In the framework of models, the relationships are variously informative, interpretive, deliberative or paternalistic, depending on whether decisions are uninfluenced, interpreted to fit the patient, deliberative to include advice or imposed from a position of superior knowledge. Third, illness-care decisions are often required at a time when the decision-making capacity of the patient is hampered, marginal or non-existent.

Much has been written about the two main alternative methods for honouring a patient's wishes in the last type of situation: that is, to elicit and honour prospective decisions as directly as possible or to designate a proxy who makes substituted or best-interests judgments. Many more fall into the greyer zones of hampered or marginal decision-making. For instance, a person recently diagnosed cancer has to make decisions in a short time frame when he or she has not had time to adjust to the significance of the diagnosis and is flooded with feelings that may blunt cognitive processing [4]. Others have illnesses, such as dementia, metabolic imbalances, brain tumours or strokes that limit one or more aspects of, or globally limit but do not eliminate, their decision-making capacity.

Decision-making near the end of life is particularly challenging. That should not be surprising: all the Achilles heels' of decision-making are particularly relevant near the end of life. Subconscious processes have a lot to say about death and dying: hormonal functions may be off due to illness; cognitive processes may also be off due to illness; the social setting is complex and powerful; people change their minds, perhaps more than ever before; and those expected to survive the patient may worry about decision-regret. However, decisions must be made.

Importantly, decision-making near the end of life also has unique features that need to be accommodated - by and with or for a person who probably has little experience of such a situation. When facing death, people make decisions about how to transition out of life that may be analogous to prior decisions (such as leaving a job or a country) or similar to decisions made by those they have seen die before them, but are usually unprecedented for the individual. As more than one dying patient has said: 'I don't know what to do; I've never died before'. At the same time, the unique features of dying are of great importance - from creating last memories, to 'showing my grandson how a real man dies with dignity' or making provisions for surviving loved ones - and decision-making needs to allow for these important features. Most of these needs transcend cultures and yet modern Western medicine has only just begun to offer suggestions for guidance [5-7].

Sadly, instead of recognizing that optimal decision-making at the end of life will always be different than optimal decision-making in other areas, some commentators have declared the area hopeless. More than one commentator has stated that advance care planning (the process of making prospective decisions) does not work because people change their minds and, anyway, they get over-ruled [8]. Indeed, it is true that problems abound. Some are inherent and relatively intractable but no better alternatives to advance planning exist. For example, anticipating future wishes is always going to be limited and yet people usually want to honour that form of autonomy as much as possible. Other problems are manageable. For instance, many people complete what advance care planning they do with a lawyer rather than their clinical team and some do not even tell their designated proxy of his or her selection for the role [9]. Policy, culture and education could remedy that situation. Overall, in the view of at least some, declarations of desuetude or uselessness are counterproductive when there is so little alternative and so much to do - which include defining and developing measures of what successful decision-making near the end of life is.

This commentary sets out six suggested directions for decision-making in this last and all important phase of every person's life. Finally, we bring these perspectives together in the form of an agenda for progress.

## Discussion

### Goals

There was a 'big thought' in the movement that was started by Louis Kutner in 1969 that it was necessary to protect patients' wishes for care near the end of life under the law through the vehicle of advance care planning. However, this was mostly obscured through the 1970s and 1980s by the focus on forms and statutes, skirmishes about whether proxy designation or written directives should be the primary vehicle for advance care planning and by turf wars about whether doctors or lawyers should be guiding (and billing for) the process. In the 1990s the Patient Self Determination Act was passed but research was underway that mired the progress with questions that were, in retrospect, poorly framed and a misguided interpretation of the findings. Modest use rates, significant change rates among choices, challenges related to the number of possible decisions patients might have to consider that might become relevant in the future and the high rate of physicians' and others' disregard of valid advance care plans discouraged many people [8]. Instead of recognizing these as either inherent or manageable problems that were less bad than the original situation, the movement stumbled.

In the meantime, the lost 'big thought' was simple and not even particularly original, but it was powerful and necessary. It was the notion that patients have their own goals for care. Since those goals do not always match the goals that had taken root in medicine - to cure illness, stamp out disease and save lives - it was also sorely needed. Patients were asking for more respect. Physicians, some of whom were startled, sometimes protested that these decisions were too complicated for untrained people to take. However, patients are real humans, real mortals and endowed with enough ability to have managed to get through life until that point (and their genes had survived the harshest of times on earth during the evolution of eras – a point which should be justification for clinicians to respect the patients' abilities). At least a substantial portion of patients understood human mortality and knew that medical interventions would one day not save them from death. This portion of patients often had different goals for care when illness arrived which had a poor prognosis. Often more than one type of goal existed in the same person at the same time. A review of the research literature identified at least six goals for palliative care patients: (1) to be cured; (2) to live longer; (3) to improve or maintain function/quality of life/independence; (4) to be comfortable; (5) to achieve life goals; and (6) to provide support for the family/caregiver [10].

Research had already demonstrated that goals predict specific treatment preferences reasonably accurately [11]. The use of goals of care seemed to be the perfect solution - and it may well still be just that. In a real sense, adherence to the patient's goal for care should define and be the gold-standard measure of quality of care near the end of life.

A body of work is beginning which could provide measures for quality care and quality of life in the seriously ill and end-of-life population. Screening instruments [12], intake evaluation instruments [13] and outcome measures [14] are all being developed in an effort to eliminate the previously mistaken assumption that life and functional ability is valued near life's end in the same way as it is during health and during chronic conditions. These developments are all to the good. Although some incorporate it, to this reviewer's knowledge, as yet no validated instrument measures the simple outcome of matching care to goals. This may partly be due to the lack of routine documentation of goals of care so that evaluations rely on personal report. However, if the patient's prime goal among a valid set of goal options (such as the above, with room for individualization) were by policy routinely documented and updated, then it would be easy to develop a method of measuring goal match.

Although there is still a frustratingly large gap between optimal and actual practice, an increasing number of clinicians do now routinely ask their patients about their goals for care and organize their care plans around the patient's priority goals. In order to ensure the translation of goals for care into physician orders that can travel with the patient from one care site to another, many are now using POLST (Physicians Orders for Life-Sustaining Treatment) or MOLST (Medical Orders for Life-Sustaining Treatment) forms or their equivalents.

### Thresholds

A critical limitation of the use of goals of care near the end of life is that goals change as the illness changes. Therefore, near the end of life, those new situations often entail a loss of decision-making capacity. So how can goals of care made in real time be useful in planning for future, different, illness situations?

The provision of scenarios to consider and the identification of goals for each scenario is one approach [15]. Then, with the strong predictive capacity of goals for treatment selection, the physician and the family will have a reasonably reliable guide based on the anticipatory decisions of the patient. Those who want to go further by illustrating and validating their goal choices by considering specific interventions can do so. If this additional step is taken, then, in order to allow for realities that cannot be anticipated, the selections should be considered as illustrative rather than binding unless the patient demands highly controlled directives. Given the uncertainty of future medical events, some researchers have found it useful to explicitly determine how much latitude the patient desires the surrogate decision maker to have when interpreting the directive in light of an unfolding clinical scenario [16].

However, articulation and recording in medical records and elsewhere of goals in various scenarios is potentially too cumbersome. So much of medicine is able to characterize an entity by a single number on a scale. Can such a simple descriptor be found in advance care planning?

One possibility is to identify the individual's threshold for switching from one type of goal to another. Usually the switch of most relevance is from curative intent to palliative intent. Preliminary work from an ambulatory care clinic population suggests that the majority of people considering their goals and treatment choices among a series of increasingly dire illness situations showed a clear threshold for that switch between curative and palliative intent, which remains relatively stable over time. Another study conducted in a nursing home population found that, for many people, the level of cognition is a key driver in defining a satisfactory quality of life and becomes an important factor for future decisions about whether to pursue further life-prolonging interventions [17].

Reasonable hope exists that a scale could be constructed on which peoples' thresholds could be codified in a concise, universal code. Such a scale could be based on illness severity - perhaps a simple ordinal scale or a compound one with several factors such as disability (incorporating cognitive components) - prognosis and treatment burden. For instance, a patient could be characterized as D9, P9, T3 meaning disability on a scale of 0-10 will be tolerated to a high level before palliative care is preferred. A poor prognosis needs to be very poor (9 on a 0-10 scale) before palliation becomes the primary goal and treatment burden is tolerable up to 3 on a 0-10 scale before palliation becomes paramount. Another patient who values function more, and is better prepared for death when the time comes, might be characterized, after answering validated questions as D3, P4, T3, and so on. The resulting scale could be used to guide decisions about specific medical interventions when an individual has reached a level of disability and prognosis deemed unsatisfactory.

### Two axes of evolving value

However, stability of preferences over time while in reasonable health is quite different from stability in the face of acute illness. Suffering drives people to do things they would not have expected and it also drives their loved ones to sink their resources into alleviating the suffering. Preferences, like those of Odysseus and women in labour, change. Presumably, that is true of goals, thresholds and specific preferences, although data on that point are limited.

Since 1993 when James Lubitz described the exponential rise in costs of care as the last year of life progresses [18], people have tried to understand who or what is driving the choices. Ultimately, most acknowledge that policy, culture and everyone involved has a hand in driving the high utilization of care near the end of life. However, the common assumption has been that this pattern is irrational. Why would anyone spend increasing amounts on diminishing returns?

Only recently have some commentators pointed out that another rational explanation could be involved. That is: the less life a person has, the more it is valued [19]. Further, the less time there is to spend what resources are available, the more it makes sense to spend whatever resources exist - unless the survivors and the next generation are being considered. For many people in the throes of suffering, altruism is harder to find. Instead the carers take on the altruistic roles and, often the resources that go beyond those of the patient also become exhausted.

People may fall into two broad categories: those for whom the increasing threat to life prompts ever more earnest attempts to hold on to it; and those for whom it prompts a letting go and a passing on of what they can to others. Research is sorely needed into this area. If these groups exist, can patients determine which group they are in? Could such identification help predict the trajectory of change, if any, of their wishes?

Until such time as research on these questions allows for a guided estimation from anticipatory choices, at least clinicians should counsel patients and their families that many people do change their preferences in the face of illness experience and that their inclination can go in either direction. As people do their advance care planning, they can take that reality into account in telling their proxy how much to allow for that type of change.

### Community standards

For those - and there will always be some - who have not engaged in discussions about goals, thresholds or any kind of care choices, there is still a helpful guiding standard. Indeed, the approach could also be helpful for those with their own directive. Studies can generate population-wide, scenario-based preferences. Then, when someone from a similar population is ill and no longer capable of decision-making, and a life-sustaining intervention choice hangs in the balance, it would at least possible to see that *x*% of people considering a similar situation would have chosen *Y*. It is similar in concept to the legal 'reasonable person' standard in which a judgment call is made about what a substitute imagined person would do; in this case the imagined person is an average population or a community member. This can be comforting to, and appreciated by, family and other decision-makers who are burdened by the decisions and the lack of any guidance from the patient's prior wishes [20].

### Roles

Many clinicians have been reminded, correctly so, to screen for depression and not to make the mistake of considering depression as 'normal' among the seriously ill. However, clinicians also have the experience of finding that people who are dying can be withdrawn and disaffected without meeting the criteria for depression. Psychiatrists and bereavement specialists from a range of disciplines know that dying does not have to be depressing even though loss is sad. It can trigger depression, but that is not the same thing. Chochinov's findings from Dignity Therapy may offer a clue to understand this phenomenon. This legacy-making intervention produces such extraordinary satisfaction and appreciation that he terms it the penicillin of palliative care, though it has minimal or no impact on depression or anxiety [21]. Perhaps interventions that the terminally ill and their families appreciate do not necessarily lift depression even when it does exist. Something else is going on.

A plausible explanation is premised on the perspective that society and modern medicine has lost track of the importance of the dying role [22]. Patients may find themselves tethered to their bed by intravenous tubing, medical routines and the isolation of medical institutions, unable to fulfill the tasks of a dying role. Perhaps what is happening to these people is that because they lack a job or role and they feel worthless or become frustrated and then withdraw because they feel unable to complete their final tasks with those they love. When Chochinov and his colleagues offer Dignity Therapy, what may be happening is that they are essentially releasing the tethers and inviting people into their last and most important role by offering to make a legacy document from their life narrative to pass on to their loved ones. The success of the activity may have to do with patients having successfully engaged in some of the tasks of their life-cycle appropriate (dying) role. Tasks of the dying role include: offering loved ones a blessing; passing on life wisdoms; sharing lasting memories through stories or small treasures or cooking recipes; settling relationships; and saying goodbye. Many of these tasks are directly or indirectly accomplished by the Dignity Therapy document that patients produce.

Social scientists guard the designation of roles carefully: criteria must be met before it can be considered a real role. Some doubt that there is such a thing as a dying role and consider that the sick role encompasses all the roles that occur around death. However, the dying role is different from the sick role, in which the person is to receive care in order to get better. Indeed, if the dying role is never entered, it may explain some of the extremes of spending near the end of life and perhaps that lack of entry into the dying role reflects the death-denying nature of present-day culture. The dying role has a life cycle stage: it has jobs or tasks; it guides people in situations where they are unsure what to do; and it involves specific relationships between people (the dying and the survivors). The dying role is best understood as being one of life's most important roles.

People care deeply about their ability to enter essential life roles: for instance, a person who wants to be a mother or a father but is unable to suffers a fate that some would consider among the worst possible. Similarly, a person who cannot be the leader or the teacher or the artist she/he felt drawn to be suffers deeply. It is reasonable to understand the disaffection and withdrawal of the dying as an expression of a similar kind of suffering: the suffering of role denial. The suffering also accrues to the bereaved. If the deceased was not able, due to a lack of guidance or opportunity, to give his or her blessing, pass on those family recipes, make a meaningful gift or make peace and pass on roles, the people who would have received those things now lack something important. Often, even among those who have lost a loved one many years ago, when they reflect on this and realize they did not receive one of those intangible, precious things, they are moved to tears.

What has this to do with clinicians and our care of the dying? Perhaps the world of medicine does not have a strong obligation to foster the dying role but it does have a strong obligation to allow for it. To impede it should be seen as an unwanted and serious side-effect of treatment. To impede it constitutes a prevention of a major source of comfort and meaning for patients and their families. Uncertainty about prognosis may pose a barrier to a smooth transition to the dying role. As was described a decade ago, many chronically ill individuals do not have a clear understanding that they are dying until the very end - in part, perhaps, because the physicians are not able to provide accurate prognostic information [23]. Similarly, physicians caring for dying nursing home residents describe the uncertainty of knowing when the patient is unlikely to benefit from further curative medical interventions [24].

One option is to improve prognostication [25]. However, clarity about when dying begins is less important than planning and preparing for death even if it may be far away. Engaging some of the tasks of the dying role can occur during youth and the years of good health. Clinicians can foster this by being comfortable with discussing the significance of the dying role with all patients and noting that it is not just for those with a poor prognosis. Whether the prognosis is days to weeks or months to years, care plans need to allow for some of the dying role tasks to be accomplished, by making space and time for visitors, for trips and shared pleasures and for interventions such as Dignity Therapy, as much as possible and as much as the patient wants. The success of a recent pilot project to move palliative care upstream - to allow palliative care to be provided alongside restorative care while demonstrating overall decreased utilization of health resources [26] - supports the possibility that some of the tasks of the dying role may be facilitated long before imminent death.

### Existential maturity: a Holy Grail or nature's basic wisdom?

Clinicians and poets watch people die and both describe how some slide 'gently into the night' and others instead depart in a firestorm of struggle [27]. In the last hours of living, some of this is probably due to pathophysiology - perhaps something to do with the state of inhibitory impulses in the central nervous system during the last hours of life [28]. However, some of it seems to antedate the last hours. Although almost surely everyone possesses a component of fighting and acceptance, it still seems that there is a general dichotomy.

Clinicians sometimes also observe that a character type seems to exist. People with this character type live their lives with a peaceful sense that every day can be enjoyed as it if were their last, they seem to live with little sense of fear and seem to die relatively peacefully. These same people may be the ones who select hospice care goals more often than curative treatment goals and they may expend less in direct care costs.

Is it possible that such people have achieved an existential maturity - a kind of peaceful acceptance of mortality and of the relationship between generations of life that mitigates the pain of our transience by allowing an understanding of how we can die without entirely ceasing to exist? Is it possible that this feature predicts end-of-life decision-making in ways that could be useful in medical care planning? Could a scale to measure such be developed?

Dylan Thomas adjured the subject of his poem [27] not to go gently into the night - likewise palliative care specialists have supported the need of some to fight for life until the last possibility has passed. It is not clear that existential maturity, in contrast to this impulse, is normative as long as life is possible. It is also clear that it can be a good and healthy thing. Most religious traditions have rituals of prayer for the ill and most have prayers that can be interpreted in such a way that, at some point, prayer for healing can be synonymous with prayer for a comfortable, natural death.

If existential maturity is a good thing for some, and if the enactment of the dying role is a good thing for both those who are dying and the bereaved, it is tempting to wonder if it could be fostered. It seems possible that enactment of tasks from the dying role foster existential maturity. Perhaps the acts of advance care planning and making a legacy document - whether it is a product of Dignity Therapy or a more home spun ethical will - foster existential maturity. Should medicine promote such enactments and, if so, to whom should they be addressed? Some high schools encourage students to write their epitaph as a way of helping them to gain a perspective of what matters in life. Perhaps such activities are fitting at every life stage, whether a person chooses the quiet or the tumultuous path to complete their life story. We do not know the answer to this: research is sorely needed because these are very important matters.

In surveys of seriously ill patients and their family members it has been found that this is what they want. Medicine has, quite rightly, concerned itself with cure. However, few people will pass along the road to death without passing through the hands of medical professionals. These medical professionals must, therefore, understand these issues and ensure that the systems of care accommodate their needs.

### An agenda for progress

Palliative care researchers need to provide us with a few conceptual advances and a few good tools to enable us to experience as good a death as possible. We need a better understanding of:

• thresholds that we require when we switch from curative to palliative intent and how these decision-drivers can be used to aide us

• our trajectory for changing the value that we place on preserving life as the remaining amount diminishes and how this decision-driver can be used to help us

• different communities' preferences for end of life care

• how the concept of the dying role can help relieve suffering and how to foster it without trying to impose it

• the concept of existential maturity.

We also need validated measures of:

• quality dying that provide at least a subscale or an independent scale which can help to match that care with the intended goals

• existential maturity.

Some of what is need is not research but the implementation of known good practices. Dissemination and implementation methods have recently received increased attention as the medical world has begun to realize that good things do not always disseminate effectively or get taken up in practice merely as a result of publication and presentation in continuing education settings. The palliative care discipline has played a leading role in the use of methods that are effective in dissemination, including through the use of quality improvement methods. More efforts are needed to close the gaps in:

• the widespread routine elicitation and use of patient goals of care among clinicians

• the use of effective advance care planning conversations

• the use of community preferences for patients for whom there is no evidence of any prior wishes.

## Conclusions

In society in general, in medicine more specifically and among individuals, much progress has been made toward the necessary level of maturity and responsible living to enable an acceptance of our mortality. However, there is still much to be done and medicine must continue to play a leading role. Through research, medicine will identify and add to the understanding of the range of wholesome paths to death and devise a way of evaluating those paths and those on them. Through training and implementation, medicine will improve the journey. It is the core of medical care, a central part of the mandate from society and our forbearers in medicine, to relieve suffering and optimize well-being in every part of the life cycle. The last phase in a person's life cycle brings a high chance of suffering and of lost critical opportunities: it can also offer the potential for important gratification and realized opportunities. As the majority of people complete this life cycle whilst in our care, the role of medicine is critical. In order to discharge our role-mandate to optimize care near the end of life, including fostering the best decisions each individual can make, medical profession must continue to undertake research and training to improve practices. We should be:

• routinely integrating goals of care discussions and documentation into our care practices

• evaluating whether personal thresholds can be used for tailored, effective advance planning

• learning more about peoples' evolving values in the face of illness

• using community standards as a reasonable guide in the absence of other information

• fostering entry into the dying role for people who have a poor prognosis

• instituting research into existential maturity.

We have come far but we still have a long way to go.

## Competing interests

The authors declare that they have no competing interests.

## Authors' contributions

LE conceived the content and drafted the manuscript. KGS critically revised the manuscript and contributed important intellectual content with regard to the discussion of thresholds and the dying role. Both authors have read and approved the final content.

## Pre-publication history

The pre-publication history for this paper can be accessed here:

http://www.biomedcentral.com/1741-7015/8/57/prepub
